# Longitudinal association of atopic dermatitis progression and keratin 6A

**DOI:** 10.1038/s41598-022-17946-x

**Published:** 2022-08-10

**Authors:** Angela Y. Zhu, Nandita Mitra, David J. Margolis

**Affiliations:** 1 Department of Biostatistics, Epidemiology, and Informatics, Perelman School of Medicine, University of Pennsylvania, 423 Guardian Drive, 108/109 Blockley Hall, Philadelphia, PA 19104 USA; 2 Department of Dermatology, Perelman School of Medicine, University of Pennsylvania, Philadelphia, PA 19104 USA

**Keywords:** Genetic association study, Skin diseases, Statistics

## Abstract

Atopic dermatitis is a common skin disease characterized by loss of skin integrity. Risk and severity have been associated with genetic variation especially with respect to the filaggrin gene, suggesting the importance of skin barrier function in atopic dermatitis pathogenesis. The keratin protein plays a role in epithelial health but its relationship with disease severity would benefit from further exploration. In this study, we evaluate the association between common keratin 6 variants and severity of atopic dermatitis over time using a Bayesian generalized linear mixed model to account for repeated measures. We identify groups of variants within which individual variants have similar effects on skin repair. Further assessment of the biological mechanisms by which these contribute to repair of epidermis may inform treatment of atopic dermatitis.

## Introduction

Atopic dermatitis (AD) is a common chronic pruritic inflammatory disease of the skin that has a prevalence of about 10–15% in children and 5–10% in adults. For many, it occurs in childhood, can wax and wane in severity, and is often associated with other illnesses like asthma and seasonal allergies^1^. The lesions tend to be erythematous with areas of skin erosion and crusting. Loss of epidermal integrity is a hallmark feature of AD, the risk for which has been associated with variation in several genes but most prominently with filaggrin, which produces a protein that is important to epidermis development^1,2^.

Variations in the genes that encode for keratin protein and in its production have been associated with many human diseases and are essential for the production and maintenance of a healthy epidermis^3,4^. Keratin 6 (KRT6), like filaggrin, is found in the outer epidermis^3,4^. KRT6 is primarily expressed in wounded or stressed keratinocytes and is used as a marker for activated keratinocytes in an unhealthy epidermis^3^. KRT6 is an alarmin that triggers the production of proinflammatory cytokines and antimicrobial peptides. KRT6 expression persists while the skin is healing and the skin barrier is improving^5^. Because of this influence of KRT6 on epithelial repair, all of these functions could be related to the severity of AD. KRT6 genetic variation has been associated with psoriasis, a chronic inflammatory disease of the skin occurring in about 1 to 4% of the population^5^. For instance, a large study on real-world comorbidities of pediatric atopic dermatitis patients found a significantly higher odds of psoriasis among those with AD as compared to controls^6^. The production of KRT6 in psoriasis is thought to be a result of T cell derived cytokines^5^. However, individuals with psoriasis are more likely to have AD than the general population^7^. Finally, individuals with pachyonychia congenita, a very rare disease, have hypertrophic nail dystrophy and very painful calluses on soles of feet and hands (keratoderma). It is associated with mutations in KRT6A, KRT6B, KRT6C, KRT16, and KRT17. More than 50% of Individuals with pachyonychia congenita have KRT6A mutations, which elicit changes to KRT6 as well as other keratin proteins. The most common KRT6A mutation (p.Asn172del) occurs in exon 9 causing stop codon two amino acids upstream from the natural stop codon^8^, and several other mutations have KRT6A been noted^9,10^.

The physical appearance of AD is often complicated by excoriation, erythema, and skin barrier breakdown. After fine-sequencing the keratin 6A (KRT6A) gene in a large longitudinal cohort of children with AD, we evaluate whether KRT6A variation is associated with the severity of AD over time and identify single nucleotide polymorphisms (SNPs) of interest.

## Methods

Patient data came from the Pediatric Eczema Elective Registry (PEER), which consisted of children who were diagnosed with atopic dermatitis^11–13^. Eligibility for the study included being between 2 and 17 years of age at enrollment and using pimecrolimus cream 1% for at least 6 weeks in the half year prior to enrollment. Every 6 months for up to 10 years, information regarding disease severity and medication use was obtained based on self-report from a questionnaire. Detailed information about medication use in this cohort has been previously published^14,15^. A subset of the cohort provided DNA samples, from which the KRT6A gene was sequenced. The study was approved by the University of Pennsylvania Institutional Review Board. All DNA samples were obtained after written informed consent was obtained, and all data were stripped of personal identifiers. Further, all methods were performed in accordance with the relevant guidelines and regulations. Since atopic dermatitis is a common illness and we sought to evaluate common alleles, the set of KRT6A SNPs that we consider are based on the a priori decision to analyze variants with minor allele frequency of at least 5%.

The primary binary outcome of disease persistence (yes/no) was measured for each participant at every six-month survey. Subjects were deemed to not have persistent AD if they reported complete disease control in the previous 6 months and were no longer using prescription creams or ointments. Our choice of outcome correlates with severity scores in both the PEER cohort^16^ and in other cohorts^17,18^. This is not a measure of time to AD remission but rather a measure of the persistence of AD at the time of assessment. Multiple measurements of the outcome of interest were available over time for each subject; the number of visits varied between 1 and 21; the average number of completed surveys was 10.1 (SD: 5.9). We used a Bayesian generalized linear mixed model (GLMM) to study the associations between SNPs of the KRT6A gene and AD persistence over time^19,20^. We included random effects in the GLMM to account for correlations between measurements on the same individual and to allow subject-specific trends. The model was used to assess the persistence of AD over the follow-up time with larger odds ratio (OR) values suggestive of lower disease persistence when the variant is present. These models were fit for the entire sample (N = 740) and then stratified by race, White (N = 393) and Black (N = 326), using the *brms* package in R^19,21^.

Specifically, for the full sample, the outcome for subject *i* at visit number *j* is modeled according to the following generalized linear mixed model with a logit link:

$$logit P\left({Y}_{ij}=1\right)= {\beta }_{0}+{b}_{0i}+{\beta }_{1}j+{b}_{1i}j+{\beta }_{2}\mathrm{variant}$$

where ${\beta }_{0}, {\beta }_{1}, \text{and} \;{\beta }_{2},$ are the intercept and regression coefficients for the fixed effects of visit number, and the variant, respectively, and ${b}_{0i}$ and ${b}_{1i}$ are the random intercept and random slope for visit number, each of which is distributed as $N\left(0,{\sigma }^{2}\right)$. For an adjusted analysis, we next fit a model that also includes the covariates age of onset, sex, and race. While age of onset and sex are not considered true confounders since they do not affect the genetic variation in individuals, we include these since they may explain some of the variability in the outcome. For the analyses stratified by race, we include age of onset and sex in the model.

For a Bayesian approach, we place priors on the fixed effect coefficients as well as the standard deviations and correlations of the random effects. Each $\beta$ is given a $N({\mathrm{0,10}}^{2})$ prior, which is a weakly informative prior to reflect absence of prior information about the effects of the variant considered. The standard deviation of subject-level effects ${\sigma }^{2}$ is given a half student-t prior with 3 degrees of freedom and a scale parameter determined by the standard deviation of the response variable after the link transformation. This choice of prior is weakly informative so that its influence is small but provides regularization for the purpose of convergence and sampling efficiency. Correlations between the random effects are given a LKJ(1) prior, so that all correlation matrices are equally likely. Our choices of noninformative priors minimize the impact of the prior relative to the data on resulting estimates. The Bayesian framework has been previously employed in transethnic meta-analysis of genomewide association studies^22,23^.

Prior to the analysis of individual SNPs, all SNPs were evaluated to determine if they were correlated. Those with high correlation, based on pairwise squared correlation coefficient (${R}^{2}$) of at least $0.70$, were combined as a single analytic unit. Table 1 gives the pairwise ${R}^{2}$ values between all the SNPs. Specifically, we see that rs177079, rs298118, rs298121, rs298122, and rs1054122 have pairwise ${R}^{2}$ values close to 1. We refer to this set as Group 1. We similarly define Groups 2, 3, and 4; the SNPs in each are provided in Table 2. Based on these groups, we create 4 composite variables that each indicate the presence of any of the SNPs in the group. For instance, the value of Composite 1 is 1 if the subject is observed to have at least one of SNPs in Group 1 and is 0 otherwise. There were three SNPs (rs376545, rs711317, rs3858631) that did not fall into any of the four groups in the sense that their pairwise correlations with all other SNPs considered had ${R}^{2}$ values less than 0.70. We then employed the Bayesian GLMM model to assess the association between each composite variable and the heal outcome.

**Table 1:** Pairwise correlations for the KRT6A SNPs with minor allele frequency of at least 5%.

	rs177079	rs188463	rs188464	rs298117	rs298118	rs298119	rs298120	rs298121	rs298122	rs376545	rs711317	rs1053854	rs1053857	rs1054122	rs1063931	rs3907935	rs4761912	rs4761913	rs11540301	rs12578949	rs12581120	rs12581781	rs17845411	rs28667515	rs61510718	rs62617088	rs1133153	rs3858630	rs3858631
rs177079	–	0.24	0.24	0.24	0.97	0.22	0.20	0.98	0.98	0.59	0.42	0.24	0.24	0.93	0.14	0.14	0.14	0.14	0.03	0.13	0.03	0.14	0.12	0.24	0.05	0.03	0.24	0.14	0.07
rs188463	–	–	0.99	0.99	0.24	0.88	0.81	0.24	0.23	0.41	0.56	0.99	0.99	0.22	0.38	0.38	0.38	0.38	0.10	0.36	0.10	0.38	0.33	0.99	0.13	0.08	0.98	0.38	0.18
rs188464	–	–	–	0.99	0.23	0.88	0.81	0.24	0.23	0.41	0.57	0.98	0.98	0.23	0.37	0.38	0.38	0.38	0.10	0.35	0.10	0.39	0.33	0.99	0.13	0.08	0.99	0.38	0.17
rs298117	–	–	–	–	0.23	0.89	0.81	0.24	0.23	0.41	0.56	0.99	0.99	0.22	0.38	0.38	0.39	0.38	0.10	0.36	0.10	0.39	0.33	0.99	0.13	0.08	0.98	0.39	0.18
rs298118	–	–	–	–	–	0.21	0.19	0.96	0.97	0.57	0.41	0.24	0.24	0.94	0.14	0.14	0.14	0.14	0.03	0.13	0.03	0.14	0.12	0.24	0.05	0.03	0.23	0.14	0.06
rs298119	–	–	–	–	–	–	0.92	0.21	0.20	0.36	0.50	0.88	0.88	0.20	0.34	0.34	0.34	0.34	0.09	0.32	0.09	0.34	0.29	0.89	0.12	0.08	0.88	0.34	0.16
rs298120	–	–	–	–	–	–	–	0.19	0.19	0.33	0.46	0.81	0.81	0.19	0.31	0.32	0.32	0.32	0.08	0.30	0.08	0.32	0.27	0.81	0.11	0.07	0.80	0.32	0.15
rs298121	–	–	–	–	–	–	–	–	0.97	0.57	0.42	0.24	0.24	0.93	0.14	0.14	0.14	0.14	0.03	0.13	0.03	0.14	0.12	0.24	0.05	0.03	0.24	0.14	0.07
rs298122	–	–	–	–	–	–	–	–	–	0.57	0.41	0.24	0.24	0.93	0.14	0.14	0.14	0.14	0.04	0.13	0.04	0.14	0.12	0.23	0.05	0.03	0.23	0.14	0.07
rs376545	–	–	–	–	–	–	–	–	–	–	0.14	0.40	0.40	0.55	0.00	0.00	0.00	0.00	0.24	0.00	0.24	0.00	0.00	0.40	0.12	0.20	0.40	0.00	0.11
rs711317	–	–	–	–	–	–	–	–	–	–	–	0.56	0.56	0.40	0.01	0.01	0.01	0.01	0.08	0.02	0.08	0.01	0.04	0.56	0.11	0.07	0.56	0.01	0.31
rs1053854	–	–	–	–	–	–	–	–	–	–	–	–	1.00	0.22	0.39	0.39	0.39	0.39	0.10	0.37	0.10	0.39	0.33	0.99	0.13	0.08	0.98	0.39	0.18
rs1053857	–	–	–	–	–	–	–	–	–	–	–	–	–	0.22	0.39	0.39	0.39	0.39	0.10	0.37	0.10	0.39	0.33	0.99	0.13	0.08	0.98	0.39	0.18
rs1054122	–	–	–	–	–	–	–	–	–	–	–	–	–	–	0.14	0.14	0.14	0.14	0.03	0.13	0.03	0.14	0.12	0.22	0.05	0.03	0.23	0.14	0.06
rs1063931	–	–	–	–	–	–	–	–	–	–	–	–	–	–	–	1.00	0.99	1.00	0.25	0.93	0.25	0.99	0.84	0.39	0.34	0.21	0.38	0.99	0.47
rs3907935	–	–	–	–	–	–	–	–	–	–	–	–	–	–	–	–	0.99	1.00	0.25	0.93	0.25	0.99	0.84	0.39	0.34	0.21	0.38	0.99	0.47
rs4761912	–	–	–	–	–	–	–	–	–	–	–	–	–	–	–	–	–	0.99	0.24	0.93	0.24	0.99	0.84	0.38	0.34	0.21	0.38	0.99	0.47
rs4761913	–	–	–	–	–	–	–	–	–	–	–	–	–	–	–	–	–	–	0.25	0.93	0.25	0.99	0.84	0.39	0.34	0.21	0.38	0.99	0.47
rs11540301	–	–	–	–	–	–	–	–	–	–	–	–	–	–	–	–	–	–	–	0.26	1.00	0.25	0.29	0.10	0.73	0.86	0.10	0.25	0.03
rs12578949	–	–	–	–	–	–	–	–	–	–	–	–	–	–	–	–	–	–	–	–	0.26	0.93	0.90	0.36	0.26	0.23	0.35	0.93	0.50
rs12581120	–	–	–	–	–	–	–	–	–	–	–	–	–	–	–	–	–	–	–	–	–	0.25	0.29	0.10	0.73	0.86	0.10	0.25	0.03
rs12581781	–	–	–	–	–	–	–	–	–	–	–	–	–	–	–	–	–	–	–	–	–	–	0.85	0.38	0.34	0.22	0.39	0.99	0.46
rs17845411	–	–	–	–	–	–	–	–	–	–	–	–	–	–	–	–	–	–	–	–	–	–	–	0.33	0.16	0.25	0.33	0.84	0.54
rs28667515	–	–	–	–	–	–	–	–	–	–	–	–	–	–	–	–	–	–	–	–	–	–	–	–	0.13	0.08	0.98	0.38	0.18
rs61510718	–	–	–	–	–	–	–	–	–	–	–	–	–	–	–	–	–	–	–	–	–	–	–	–	–	0.63	0.13	0.34	0.04
rs62617088	–	–	–	–	–	–	–	–	–	–	–	–	–	–	–	–	–	–	–	–	–	–	–	–	–	–	0.08	0.22	0.02
rs1133153	–	–	–	–	–	–	–	–	–	–	–	–	–	–	–	–	–	–	–	–	–	–	–	–	–	–	–	0.38	0.17
rs3858630	–	–	–	–	–	–	–	–	–	–	–	–	–	–	–	–	–	–	–	–	–	–	–	–	–	–	–	–	0.47
rs3858631	–	–	–	–	–	–	–	–	–	–	–	–	–	–	–	–	–	–	–	–	–	–	–	–	–	–	–	–	–

**Table 2:** Estimates of the effect (odds ratio) of each correlated group on AD disease persistence and corresponding posterior intervals. OR greater than 1 indicate less disease persistence.

	All				Whites		Blacks	
	Unadjusted		Adjusted		Adjusted		Adjusted	
	OR	95% CrI	OR	95% CrI	OR	95% CrI	OR	95% CrI
Composite 1	2.12	[1.32, 3.39]*	1.70	[1.11, 2.69]*	1.35	[0.80, 2.29]	1.57	[0.66, 3.90]
Composite 2	0.58	[0.37, 0.92]*	0.63	[0.41, 0.97]*	0.64	[0.37, 1.13]	0.80	[0.37, 1.70]
Composite 3	0.41	[0.23, 0.73]*	0.63	[0.33, 1.15]	0.82	[0.16, 4.22]	0.75	[0.35, 1.62]
Composite 4	1.20	[0.66, 2.29]	1.19	[0.64, 2.32]	1.03	[0.48, 2.12]	0.84	[0.29, 2.29]
**Others**								
rs376545	1.51	[1.08, 2.10]*	1.46	[1.06, 1.99]*	1.27	[0.85, 1.88]	1.27	[0.73, 2.25]
rs711317	1.63	[1.22, 2.20]*	1.34	[0.94, 1.84]	1.06	[0.72, 1.52]	1.30	[0.70, 2.46]
rs3858631	0.90	[0.61, 1.32]	0.77	[0.52, 1.19]	0.70	[0.43, 1.16]	0.90	[0.38, 2.03]

Composite variables are based on presence of at least one SNP in each correlated group:

Group 1 = [rs177079, rs298118, rs298121, rs298122, rs1054122].

Group 2 = [rs1063931, rs3907935, rs4761912, rs4761913, rs12578949, rs12581781, rs17845411, rs3858630].

Group 3 = [rs11540301, rs12581120, rs61510718, rs62617088].

Group 4 = [rs188463, rs188464, rs298117, rs298119, rs298120, rs1053854, rs1053857, rs28667515, rs1133153].

*Credible interval (CrI) is either completely above or below 1.

## Results

Table 2 provides the associations of SNP groups as well as individual SNPs with AD persistence over time based on odds ratio (OR) and corresponding 95% credible intervals. For each composite variable or variant, an OR greater than 1 indicates that the odds of skin healing are greater for subjects with that variant while an OR less than 1 indicates the variant is associated with persistence of atopic dermatitis. The interpretation of the 95% credible interval is that, given observed data, the probability that the true OR estimate lies within the interval is 0.95; that is, it provides the range that contains the middle 95% of probable OR values based on the posterior distribution. Variants with credible intervals that do not contain 1 are deemed to be significantly associated with skin healing. As the premise of Bayesian methods considers the data or evidence to be fixed and are contingent on the available information, multiple testing corrections are not applicable^24^.

Subjects in Group 1 have approximately twice the odds of healing over time as compared to those that do not have any of those SNPs. The effect is in the opposite direction for Composites 2 and 3 as subjects have significantly greater AD disease persistence (Table 2). On the other hand, there does not appear to be an association between having the variants in Group 4 and disease persistence over time. rs376545 and rs711317 also have 95% credible intervals for the OR estimate that lay completely above 1, which suggest that these variants may result in improved skin healing over time. Further, the stratified estimates tend to be in the same direction as the overall estimates—that is, the effects of the SNPs on skin repair are similar for Whites and Blacks, although results were not statistically significant. Individual SNP associations are reported in Supplementary Table 1.

## Discussion

In this study, we consider Bayesian generalized linear mixed models to evaluate the relationship between KRT 6A variants and atopic dermatitis outcomes and disease progression. These models have been commonly employed in RNA sequencing and microarray studies and artificial intelligence pipelines^25^. By utilizing a Bayesian approach for our analyses, the corresponding interpretation focuses on effect estimates and credible intervals rather than hypothesis testing which relies on significance levels and p-values.

SNPs in Group 1 were found to be associated with greater healing and repair of the skin; these are likely to assist with wound repair and re-epithelization. On the other hand, SNPs in Groups 2 and 3 resulted in worse skin outcomes, so healing may require longer duration. Building on present understanding for keratinocyte’s role in AD development^26^, further study into the mechanisms by which certain KRT6A SNPs affect keratinocyte activity and corresponding immune responses as well as interactions with treatments are warranted. As keratinocytes mediate immunity, greater exploration of the biological processes could provide insight into AD progression and therapeutic efforts.

## Supplementary Information

Supplementary Information.

## Data Availability

The data set related to this article can be found at 10.17632/bg4bvx2g7p.1, hosted at Mendeley Data (Zhu, Yaqian (2021), “KRT6A SNP and AD Longitudinal Data”, Mendeley Data, V1, 10.17632/bg4bvx2g7p.1).

## References

[1] Weidinger S, Beck LA, Bieber T, Kabashima K, Irvine AD (2018). Atopic dermatitis. Nat. Rev. Dis. Primers.

[2] DavidBoothe W, Tarbox JA, Tarbox MB (2017). Atopic dermatitis: pathophysiology. Adv. Exp. Med. Biol..

[3] Freedberg IM, Tomic-Canic M, Komine M, Blumenberg M (2001). Keratins and the keratinocyte activation cycle. J. Investig. Dermatol..

[4] Haines RL, Lane EB (2012). Keratins and disease at a glance. J. Cell Sci..

[5] Zhang X, Yin M, Zhang L-J (2019). Keratin 6, 16 and 17-critical barrier alarmin molecules in skin wounds and psoriasis. Cells.

[6] Huang AH (2021). Real-world comorbidities of atopic dermatitis in the pediatric ambulatory population in the United States. J. Am. Acad. Dermatol..

[7] Dai Y-X, Tai Y-H, Chang Y-T, Chen T-J, Chen M-H (2021). Bidirectional association between psoriasis and atopic dermatitis: a nationwide population-based cohort study. Dermatology.

[8] Wilson NJ (2012). Homozygous dominant missense mutation in keratin 17 leads to alopecia in addition to severe pachyonychia congenita. J. Investig. Dermatol..

[9] Spaunhurst KM (2012). Pachyonychia congenita patients with mutations in KRT6A have more extensive disease compared with patients who have mutations in KRT16. Br. J. Dermatol..

[10] Forrest CE, Casey G, Mordaunt DA, Thompson EM, Gordon L (2016). Pachyonychia congenita: a spectrum of KRT 6a mutations in Australian patients. Pediatr. Dermatol..

[11] Kapoor R (2008). The prevalence of atopic triad in children with physician-confirmed atopic dermatitis. J. Am. Acad. Dermatol..

[12] Margolis DJ (2019). Association of filaggrin loss-of-function variants with race in children with atopic dermatitis. JAMA Dermatol..

[13] Margolis DJ (2012). The persistence of atopic dermatitis and filaggrin (FLG) mutations in a US longitudinal cohort. J. Allergy Clin. Immunol..

[14] Berna R, Margolis DJ, Barbieri JS (2021). Attrition of topical calcineurin inhibitor use over time in patients with atopic dermatitis. J. Am. Acad. Dermatol..

[15] Kapoor R, Hoffstad O, Bilker W, Margolis DJ (2009). The frequency and intensity of topical pimecrolimus treatment in children with physician-confirmed mild to moderate atopic dermatitis. Pediatr. Dermatol..

[16] Chang J, Bilker W, Hoffstad O, Margolis D (2017). Cross-sectional comparisons of patient-reported disease control, disease severity and symptom frequency in children with atopic dermatitis. Br. J. Dermatol..

[17] Silverberg JI (2018). Severity strata for POEM, PO-SCORAD, and DLQI in US adults with atopic dermatitis. Ann. Allergy Asthma Immunol..

[18] Silverberg JI (2018). Content and construct validity, predictors, and distribution of self-reported atopic dermatitis severity in US adults. Ann. Allergy Asthma Immunol..

[19] Bürkner P-C (2017). brms: An R package for Bayesian multilevel models using stan. J. Stat. Softw..

[20] Liang K-Y, Zeger SL (1986). Longitudinal data analysis using generalized linear models. Biometrika.

[21] R Core Team. *R: A language and environment for statistical computing*. (2021).

[22] Li YR, Keating BJ (2014). Trans-ethnic genome-wide association studies: advantages and challenges of mapping in diverse populations. Genome Med..

[23] Morris AP (2011). Transethnic meta-analysis of genomewide association studies. Genet. Epidemiol..

[24] Gelman A, Hill J, Yajima M (2009). Why we (usually) don’t have to worry about multiple comparisons. J. Res. Educ. Eff..

[25] Vestal BE (2020). MCMSeq: Bayesian hierarchical modeling of clustered and repeated measures RNA sequencing experiments. BMC Bioinform..

[26] Chieosilapatham P (2021). Keratinocytes: Innate immune cells in atopic dermatitis. Clin. Exp. Immunol..

