# Supplementary material for: Longitudinal association of atopic dermatitis progression and keratin 6A
Source: Sci Rep. 2022 Aug 10;12:13629. doi: 10.1038/s41598-022-17946-x (PMC9365824; doi:10.1038/s41598-022-17946-x)
Supplement: Supplementary file 1 — Supplementary Information. [file 41598_2022_17946_MOESM1_ESM.pdf]

Supplementary Materials for

Longitudinal association of atopic dermatitis progression and keratin 6A

in *Scientific Reports*

Angela Y. Zhu (0000-0001-5187-2667)[1*], Nandita Mitra (0000-0002-7714-3910)[1], David J. Margolis (0000-0002-0506-8085)[1,2]

[1]Department of Biostatistics, Epidemiology, and Informatics, Perelman School of Medicine;
[2]Department of Dermatology, Perelman School of Medicine; University of Pennsylvania, Philadelphia, PA 19104, USA.

[*]Corresponding author

Estimates of association for individual SNPs

Supplementary Table 1. Estimates of the effect (odds ratio) of each correlated group and individual variants on the heal outcome and corresponding posterior intervals.

		All				Whites		Blacks	
		Unadjusted		Adjusted		Adjusted		Adjusted	
		OR	95% CrI	OR	95% CrI	OR	95% CrI	OR	95% CrI
Composite 1		2.12	[1.32, 3.39]*	1.70	[1.11, 2.69]*	1.35	[.80, 2.29]	1.57	[.66, 3.90]
	rs177079	1.92	[1.36, 2.72]*	1.63	[1.19, 2.27]*	1.32	[.87, 1.97]	1.55	[.68, 3.46]
	rs298118	1.97	[1.43, 2.69]*	1.72	[.83, 2.46]	1.42	[.93, 2.18]	1.51	[.65, 3.06]
	rs298121	1.92	[1.35, 2.72]*	1.64	[1.13, 2.36]*	1.35	[.90, 1.99]	1.45	[.61, 3.25]
	rs298122	1.88	[1.36, 2.59]*	1.60	[1.15, 2.25]*	1.31	[.86, 1.92]	1.48	[.64, 3.42]
	rs1054122	2.05	[1.45, 2.92]*	1.75	[1.23, 2.53]*	1.48	[.97, 2.16]	1.34	[.58, 2.97]
Composite 2		.58	[.37, .92]*	.63	[.41, .97]*	.64	[.37, 1.13]	.80	[.37, 1.70]
	rs1063931	.68	[.47, .94]*	.76	[.54, 1.06]	.68	[.42, 1.07]	.97	[.56, 1.63]
	rs3907935	.68	[.48, .96]*	.76	[.53, 1.07]	.68	[.44, 1.07]	.98	[.60, 1.75]
	rs4761912	.68	[.49, .98]*	.78	[.54, 1.12]	.68	[.43, 1.07]	1.02	[.60, 1.75]
	rs4761913	.68	[.48, .96]*	.76	[.53, 1.07]	.68	[.42, 1.13]	.97	[.56, 1.58]
	rs12578949	.70	[.50, .98]*	.77	[.54, 1.09]	.65	[.40, 1.05]	.97	[.56, 1.65]
	rs12581781	.69	[.50, .99]*	.77	[.54, 1.07]	.68	[.43, 1.08]	.98	[.58, 1.63]
	rs17845411	.76	[.52, 1.08]	.82	[.57, 1.15]	.68	[.41, 1.13]	1.05	[.62, 1.80]
	rs3858630	.69	[.50, .94]*	.77	[.55, 1.11]	.70	[.44, 1.14]	1.00	[.59, 1.68]
Composite 3		.41	[.23, .73]*	.63	[.33, 1.15]	.82	[.16, 4.22]	.75	[.35, 1.62]
	rs11540301	.63	[.38, 1.04]	.90	[.49, 1.62]	.48	[.08, 2.44]	1.05	[.56, 1.93]
	rs12581120	.61	[.37, 1.00]	.90	[.49, 1.62]	.51	[.08, 2.83]	1.07	[.57, 1.97]
	rs61510718	.58	[.36, .92]*	.84	[.51, 1.38]	.79	[.16, 3.97]	1.01	[.58, 1.75]
	rs62617088	.62	[.37, 1.08]	.89	[.49, 1.57]	.46	[.07, 2.69]	1.01	[.54, 2.01]
Composite 4		1.20	[.66, 2.29]	1.19	[.64, 2.32]	1.03	[.48, 2.12]	.84	[.29, 2.29]
	rs188463	1.30	[.95, 1.80]	1.21	[.88, 1.67]	1.06	[.71, 1.52]	1.15	[.67, 1.97]
	rs188464	1.32	[.96, 1.84]	1.21	[.85, 1.67]	1.07	[.73, 1.57]	1.19	[.72, 2.01]
	rs298117	1.31	[.96, 1.84]	1.21	[.87, 1.65]	1.04	[.68, 1.54]	1.15	[.68, 1.99]
	rs298119	1.11	[.82, 1.54]	1.06	[.78, 1.48]	1.05	[.70, 1.57]	.86	[.49, 1.42]
	rs298120	1.08	[.78, 1.49]	1.09	[.78, 1.54]	1.07	[.73, 1.57]	.90	[.53, 1.55]
	rs1053854	1.27	[.93, 1.79]	1.19	[.86, 1.63]	1.02	[.68, 1.55]	1.16	[.72, 1.92]
	rs1053857	1.27	[.94, 1.75]	1.19	[.86, 1.65]	1.03	[.68, 1.52]	1.15	[.68, 1.92]
	rs28667515	1.27	[.91, 1.77]	1.19	[.88, 1.63]	1.02	[.68, 1.52]	1.16	[.69, 1.92]
	rs1133153	1.27	[.90, 1.73]	1.21	[.87, 1.70]	1.04	[.68, 1.54]	1.17	[.71, 1.99]
Others									
	rs376545	1.51	[1.08, 2.10]*	1.46	[1.06, 1.99]*	1.27	[.85, 1.88]	1.27	[.73, 2.25]
	rs711317	1.63	[1.22, 2.20]*	1.34	[.94, 1.84]	1.06	[.72, 1.52]	1.30	[.70, 2.46]
	rs3858631	.90	[.61, 1.32]	.77	[.52, 1.19]	.70	[.43, 1.16]	.90	[.38, 2.03]

** Credible interval (CrI) is either completely above or below 1.*
